# Outcomes of Liver Resection for Metabolic Dysfunction-Associated Fatty Liver Disease or Chronic Hepatitis B-Related HCC

**DOI:** 10.3389/fonc.2021.783339

**Published:** 2022-01-20

**Authors:** Lei Liu, Si Xie, Yu-Xian Teng, Zhu-Jian Deng, Kang Chen, Hao-Tian Liu, Rong-Rui Huo, Xiu-Mei Liang, Ping-Ping Guo, Da-Long Yang, Liang Ma, Bang-De Xiang, Le-Qun Li, Jian-Hong Zhong

**Affiliations:** ^1^ Hepatobiliary Surgery Department, Fourth Affiliated Hospital of Guangxi Medical University, Liuzhou Workers Hospital, Liuzhou, China; ^2^ Hepatobiliary Surgery Department, Guangxi Liver Cancer Diagnosis and Treatment Engineering and Technology Research Center, Guangxi Medical University Cancer Hospital, Nanning, China

**Keywords:** chronic hepatitis B, hepatocellular carcinoma (HCC), liver resection, metabolic dysfunction-associated fatty liver disease, overall survival

## Abstract

**Aims:**

This study aims to determine differences in severity of background liver disease at hepatocellular carcinoma (HCC) diagnosis and long-term survival outcomes among patients undergoing liver resection for HCC in the background of metabolic dysfunction-associated fatty liver disease (MAFLD) compared to chronic hepatitis B (CHB) alone or concurrent CHB (CHB/MAFLD).

**Methods:**

Patient demographics and comorbidities, clinicopathologic data, perioperative and long-term outcomes among patients who underwent liver resection for HCC were reviewed. Overall and recurrence-free survival were calculated with the Kaplan-Meier method, with the values compared using the log-rank test.

**Results:**

From January 2014 to December 2018, 1325 patients underwent potential curative liver resection of HCC; 67 (5.0%), 176 (13.3%), and 1082 (81.7%) patients had MAFLD alone, CHB concurrent with MAFLD, and CHB alone, respectively. At HCC diagnosis, fewer MAFLD patients had cirrhosis, alpha fetoprotein concentration ≥ 400 ng/mL, tumor size ≥ 5 cm, mulinodular, microvascular invasion, receiving major hepatectomy, and receiving adjuvant transarterial chemoembolization. After a median follow-up of 47 months after liver resection, MAFLD (or MAFLD plus CHB/MAFLD) patients had significantly higher overall and recurrence-free survival than CHB patients before or after propensity score analysis (all *P*<0.05).

**Conclusion:**

Patients with HCC in the setting of MAFLD have less-severe background liver disease at HCC diagnosis and better long-term survival after curative liver resection compared to counterparts with CHB/MAFLD or CHB.

## Introduction

Hepatocellular carcinoma (HCC) is the sixth most common malignancy and the third leading cause of cancer-related death (1), with East Asia demonstrating the highest incidence of HCC worldwide (2). Past epidemiological data suggest that hepatitis B virus (HBV), hepatitis C virus (HCV), and alcohol consumption are three predominant causative factors of HCC. In recent decade, nonalcoholic fatty liver disease (NAFLD) is already a rapidly increasing risk factor of HCC in the USA, France and the UK (3, 4). The estimated annual incidence of HCC among patients with NAFLD is lower than that among those with HBV or HCV infection (5, 6). However, it is estimated that 25% of the global population have NAFLD, with the highest prevalence in high income regions (6). Namely, more worldwide people have NAFLD than other liver diseases, such as HBV or HCV infection, leading the necessary to analyze the prognoses of patients with NAFLD-related HCC.

NAFLD is defined as excess hepatic fat accumulation (>5%) after the exclusion of significant alcohol consumption or any other causes of steatosis, such as certain toxins and drugs (7–10). Metabolic dysfunction-associated fatty liver disease (MAFLD), formerly named NAFLD, is a chronic disease characterized by fat accumulation in the liver with an underlying metabolic dysregulation, for which there is no approved pharmacotherapy (10). Recently, several official guidelines and consensus provide simple and practical diagnostic criteria for the disease (10–12). MAFLD more closely implies the presence of type 2 diabetes mellitus, overweight/obesity, or metabolic dysregulation, contributing to better identification of individuals with this metabolic liver disease (13). More importantly, MAFLD may eventually progress to liver-related complications, including HCC. A populational cohort from Switzerland found the burden of NAFLD- and MAFLD-related HCCs significantly increased over the years, particularly in women (14).

Liver resection is one of the main curative treatments for patients with HCC (15, 16). However, HCC recurrence after liver resection is high. Systematic reviews with large sample size revealed that the 5-years recurrence-free survival are 37%, 25%, and 18% in patients with early, intermediate, or advanced disease, respectively (16, 17). And the corresponding 5-years overall survival are 67%, 30%, and 18%, respectively (16, 17). Tumor stage and treatment measures are the most important factors affecting the prognosis of patients with HCC. However, our understanding of the association between etiological factors and the prognoses of patients with HCC after liver resection is limited. Furthermore, the contribution of MAFLD to the HCC burden according to clinical and tumor characteristics remain unclear. We therefore aimed to compare the clinical and tumor characteristics and the outcomes of patients with HBV- or MAFLD-related HCC after liver resection.

## Patients and Methods

### Study Design and Population

This was a retrospective study. All patients with a diagnosis of HCC based on postoperative histopathology between 1 January 2014 and 31 December 2018 might be eligible for the study. Consecutive patients with HCC were identified *via* the electronic medical records of Guangxi Medical University Cancer Hospital, Nanning, China, and the Fourth Affiliated Hospital of Guangxi Medical University, Liuzhou, China. Patients were selected based on the following eligibility criteria: (1) patients were without preoperative neoadjuvant therapy (including transarterial chemoembolization, radiotherapy, sorafenib, lenvatinib, immune checkpoint inhibitors, etc), (2) had primary HCC and underwent potential curative liver resection (macroscopically tumor-free), (3) diagnoses of HCC were confirmed by postoperative histopathology, and (4) suffered from chronic hepatitis B (CHB) or MAFLD. Patients were excluded if they were with (1) intrahepatic cholangiocarcinoma, (2) positive anti-HCV or human immunodeficiency virus, (3) alcohol consumption, (4) autoimmune hepatitis, (5) drug-induced liver injury, (6) combined with other malignancies, or (7) incomplete medical information. All the procedures were carried out in accordance with the Helsinki Declaration of 1975. Written informed consent was not provided by the patients because this was a retrospective study. After obtaining institutional board review approval from Guangxi Medical Cancer Hospital (LW2021042) and the Fourth Affiliated Hospital of Guangxi Medical University (LW2021005), demographics, comorbid conditions, clinicopathologic data, radiology reports, and long-term outcomes of the included patients were reviewed.

Based on the aim of the study, included patients would be divided into three groups, namely, patients with MAFLD-related HCC (MAFLD group), patients with CHB-related HCC (CHB group), and those with dual etiology related HCC (CHB/MAFLD group).

### Liver Resection

The indications of liver resection were based on the Chinese guideline for HCC (18). Patients should be with Child-Pugh score of no more than 7. The presence of appropriate residual liver volume was determined by volumetric dynamic enhanced computerized tomography or magnetic resonance imaging (MRI). The selection criteria and procedures of liver resection have been detailed elsewhere (19–21). The gallbladder was routinely excised before liver resection. Adequate drainage was routinely monitored. Intraoperative ultrasound was performed when necessary.

### Definition

The diagnosis of MAFLD was based on hepatic steatosis [detected either by imaging techniques (ultrasound, computerized tomography or MRI) or by postoperative liver histopathology] in addition to overweight/obesity (body mass index ≥23 kg/m^2^), presence of type 2 diabetes mellitus, or evidence of metabolic dysregulation (10, 11). Hepatic steatosis and cirrhosis were determined directly by imaging or histopathology reports. Fibrosis-4, waist circumference, insulin resistance score, plasma high-sensitivity C-reactive protein level, or blood biomarkers/scores of MAFLD was not assessed in this study. Alcohol consumption was defined as excessive alcohol intake (>30 g/day in men, >20 g/day in women) (22). In this study, dual etiology of HCC was defined as a patient suffered from both CHB and MAFLD (23). CHB was defined as the presence of positive of hepatitis B surface antigen HBV DNA, and/or hepatitis B core antibody. CHB-related HCC was defined as a patient with HCC accompanied by CHB. Information on alcohol consumption, autoimmune hepatitis, and drug-induced liver injury was obtained from the past medical history or personal history of medical records. Major hepatectomy was defined as the resection of three or more Couinaud segments (24).

### Data Collection and Outcomes

The number or level of the following data were collected and analyzed *via* the electronic medical records: gender, age, body mass index, hypertension, type 2 diabetes mellitus, hepatic steatosis, liver cirrhosis, Child-Pugh liver function grade, tumor size, tumor number, macrovascular invasion, Barcelona Clinical Liver Cancer stage, hepatitis B surface antigen, alpha fetoprotein, alanine aminotransferase, aspartate aminotransferase, plasma triglycerides, high density lipoprotein cholesterol, total bilirubin, albumin, prealbumin, microvascular invasion, resection type, and adjuvant transarterial chemoembolization.

The primary outcome was overall survival, calculated from the date of liver resection to the date of death from any cause or the date of the last follow-up. No patient undergone live transplantation in this cohort. Follow-up data of overall survival was obtained from the hospital database (X.-M.L). The secondary endpoints included recurrence-free survival and perioperative mortality and morbidity. Recurrence-free survival was calculated from the date of liver resection to the date of tumor recurrence or death from any cause, whichever occurred first. HCC recurrence, which was assessed by the investigators (J.-H.Z, Y.-X.T, K.C), was diagnosed using enhanced computed tomography and/or MRI [including gadolinium-ethoxybenzyl-diethylenetriamine pentaacetic acid (Gd-EOB-DTPA)-enhanced MRI] with or without alpha fetoprotein ≥400 ng/mL) (18). Patients with HCC recurrence would be received appropriate therapeutic approaches, with radiofrequency ablation and repeat liver resection as the preferred curative treatments (25, 26). Postoperative mortality was defined as the rate of death within 30 days after liver resection. Perioperative morbidity was graded according to the Clavien-Dindo classification (27). Clavien-Dindo grade of at least 3a was defined as major morbidity. The follow-up period was up to March 2021 or death. Patients with CHB routinely received antiviral therapy with nucleos(t)ide analogue (28). Moreover, patients with high risk factors of HCC recurrence, such as tumor size at least 5 cm, multinodules, involving macrovascular invasion or microvascular invasion, would receive adjuvant transarterial chemoembolization (29, 30). However, patients with MAFLD did not receive corresponding treatment for MAFLD.

### Statistical Analysis

Categorical variables were expressed as number and percentage and compared with Pearson’s chi-square tests. Bonferroni test was used for multiple comparisons. Continuous variables were expressed as mean ± standard deviation and compared by using the one-way analysis of variance for normal distributions. For skewed distributions, variables were expressed as median (interquartile range) and compared by using the Kruskal-Wallis test. Overall and recurrence-free survival were calculated with the Kaplan-Meier method, with the values compared using the log-rank test. To reveal the association between different counterparts (MAFLD, CHB/MAFLD, and CHB) and overall or recurrence-free survival after liver resection, multivariate analyses were performed using a Cox proportional hazards model. Any confounders that a change in effect estimate was more than 5% or recognized to be associated with the outcomes were entered into a Cox regression analysis. A two-tailed *p* value < 0.05 was considered to indicate statistically significant. GraphPad Prism 8.0 and IBM SPSS (ver. 26.0 SPSS Inc., Chicago, IL, USA) softwares were used to perform the analyses.

To reduce the potential effect of baseline variable imbalance on prognosis, propensity score matching between groups at a 1:3 ratio was used. All collected variables were involved in propensity score matching except body mass index, hypertension, type 2 diabetes mellitus, triglycerides, and high density lipoprotein cholesterol, which were used to define MAFLD. The propensity score was generated by a logistic regression. Nearest-neighbor caliper matching without replacement (random order or closest distance) was used to pair MAFLD (or MAFLD plus CHB/MAFLD) and CHB patients with similar propensity score values (31).

## Results

### Patient Demographic and Baseline Clinical Characteristics

**Figure 1:**
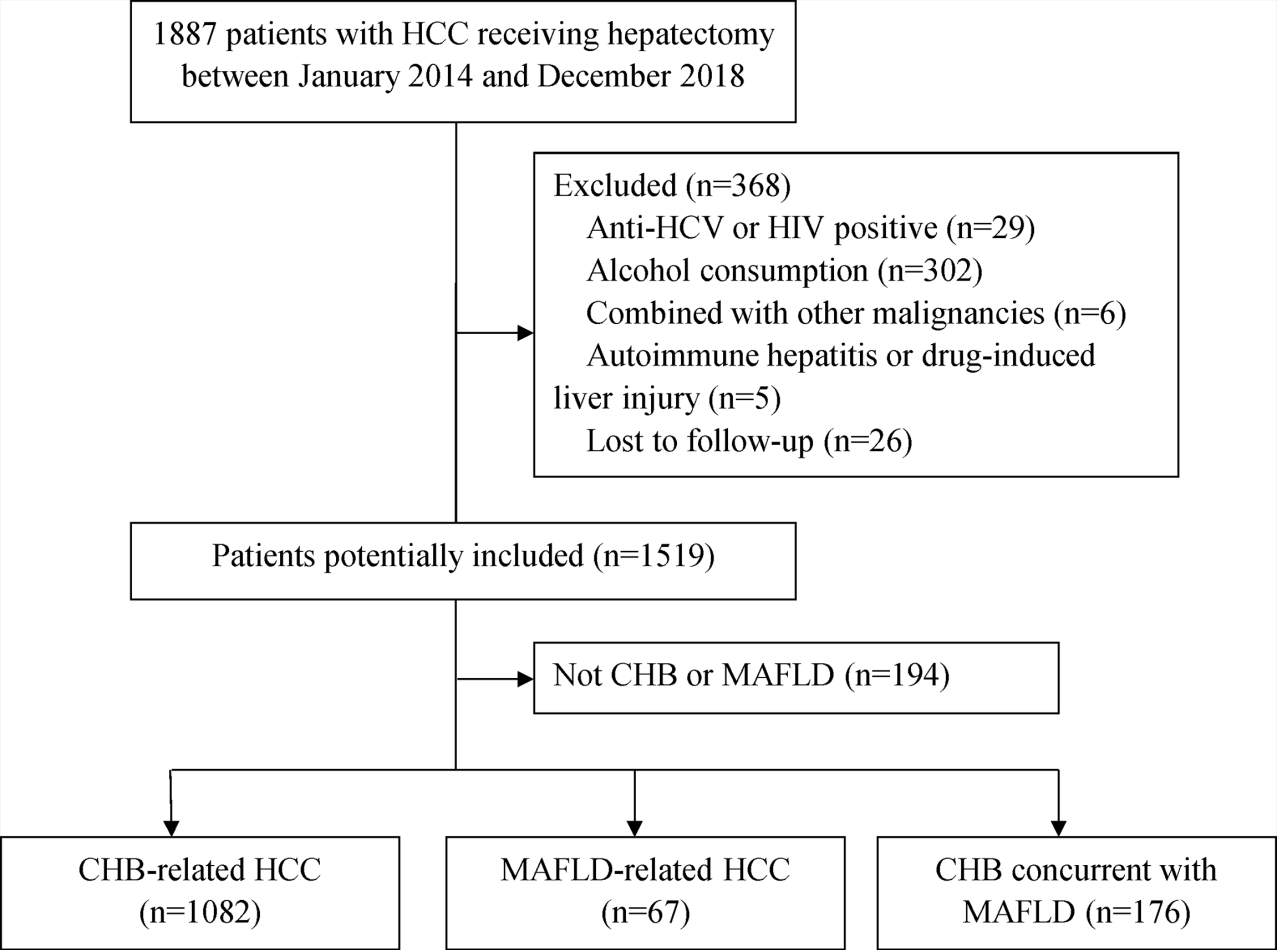
Patient selection flow. CHB, chronic hepatitis B; HCC, hepatocellular carcinoma; HCV, hepatitis C virus; HIV, human immunodeficiency virus; MAFLD, metabolic dysfunction-associated fatty liver disease.

A total of 1887 patients underwent potential curative liver resection of HCC from January 2014 from December 2018; 368 had with anti-HCV or HIV positive, alcohol consumption, combined with other malignancies, autoimmune hepatitis or drug-induced liver injury and were excluded from this study. Other 194 patients without CHB or MAFLD were also excluded. Of the remaining 1325 patients, 67 (5.0%) had MAFLD alone, 1082 (81.7%) had CHB alone, and 176 (13.3%) had CHB concurrent with MAFLD (**Figure 1**). The diagnosis of hepatic steatosis was detected by imaging techniques alone (13, 3.8%), liver histopathology alone (299, 88.2%) or confirmed by both (27, 8.0%).

Compared to patients with CHB, MAFLD patients were older (58.8 *vs* 48.9 yrs), more frequently had overweight/obesity (62.7% *vs* 33.3%), hypertension (35.8% *vs* 6.4%), type 2 diabetes mellitus (26.9% *vs* 5.8%), and early stage disease (77.6% *vs* 59.0%, BCLC stage 0/A). Hepatic synthetic function at HCC presentation (measured by prothrombin time, prealbumin, alanine aminotransferase, and aspartate aminotransferase levels) was better in MAFLD patients. There were no differences in rates of gender, smoking, Child-Pugh grade B, or macrovascular invasion. Moreover, fewer MAFLD patients had cirrhosis (50.7% *vs* 71.3%), preoperative alpha fetoprotein concentration ≥ 400 ng/mL (23.9% *vs* 42.2%), tumor size ≥ 5 cm (49.3% *vs* 61.6%), mulinodular (10.4% *vs* 22.5%), microvascular invasion (41.8% *vs* 56.7%), and receiving major hepatectomy (19.4% *vs* 34.1%) or adjuvant transarterial chemoembolization (32.8% *vs* 55.0%) than CHB patients. However, MAFLD patients had higher level of triglycerides (**Table 1**).

**Table 1:** Patient baseline demographic and clinical characteristics.

Variables	MAFLD, n=67 (%)	CHB/MAFLD, n=176 (%)	CHB, n=1082 (%)	*P* value		
				MAFLD *vs* CHB	MAFLD *vs* CHB/MAFLD	CHB/MAFLD *vs* CHB
Gender, female	10 (14.9)	21 (11.9)	151 (14.0)	0.824	0.532	0.469
Age, year	58.8 ± 10.0	49.4 ± 9.4	48.9 ± 10.8	<0.001	<0.001	0.537
Body mass index, ≥23 kg/m^2^	42 (62.7)	166 (94.3)	360 (33.3)	<0.001	<0.001	<0.001
Smoking, present	20 (29.9)	56 (31.8)	408 (37.7)	0.197	0.768	0.133
Hypertension, present	24 (35.8)	29 (16.5)	69 (6.4)	<0.001	0.001	<0.001
Type 2 diabetes mellitus, present	18 (26.9)	30 (17.0)	63 (5.8)	<0.001	0.086	<0.001
Hepatic steatosis, present	67 (100)	176 (100)	96 (8.9)	<0.001	1.000	<0.001
Liver cirrhosis, present	34 (50.7)	120 (68.2)	771 (71.3)	<0.001	0.012	0.405
Child-Pugh grade B	4 (6.0)	5 (2.8)	83 (7.7)	0.610	0.439	0.020
Triglycerides, mmol/L	1.3 (0.9, 1.8)	0.9 (0.9, 1.4)	0.9 (0.7, 1.1)	<0.001	0.041	<0.001
High-density lipoprotein, mmol/L	1.2 ± 0.4	1.2 ± 0.3	1.21 ± 0.34	0.314	0.317	0.941
Alpha fetoprotein, ≥400 ng/ml	16 (23.9)	51 (29.0)	457 (42.2)	0.003	0.427	0.001
Platelet count, <100 x10^9^/L	1 (1.5)	8 (4.5)	88 (8.1)	0.048	0.456	0.096
Prothrombin time	12.2 ± 0.9	12.8 ± 1.3	12.9 ± 1.4	<0.001	<0.001	0.085
Total bilirubin, μmol/L	13.4 (9.9, 18.4)	13.8 (10.6, 17.4)	13.6 (10.1, 18.3)	0.846	0.793	0.925
Albumin, g/L	39.6 ± 4.2	39.6 ± 4.1	38.9 ± 4.7	0.220	0.969	0.048
Prealbumin, g/L	221.3 ± 66.5	195.2 ± 57.5	172.9 ± 66.3	<0.001	0.003	<0.001
Alanine aminotransferase, U/L	26.0 (18.5, 35.0)	40.0 (27.0, 56.0)	37.0 (26.0, 54.0)	<0.001	<0.001	0.122
Aspartate aminotransferase, U/L	28.0 (23.0, 36.5)	37.0 (30.0, 50.0)	41.0 (31.0, 61.0)	<0.001	<0.001	0.006
Tumor size, >5 cm	33 (49.3)	96 (54.5)	667 (61.6)	0.044	0.460	0.074
Tumor number, multiple	7 (10.4)	44 (25.0)	243 (22.5)	0.021	0.013	0.456
Macrovascular invasion, present	7 (10.4)	21 (11.9)	197 (18.2)	0.107	0.746	0.041
BCLC stage				0.010	0.013	0.008
0/A	52 (77.6)	106 (60.2)	638 (59.0)			
B	6 (9.0)	45 (25.6)	196 (18.1)			
C	9 (13.4)	25 (14.2)	248 (22.9)			
Major hepatectomy	13 (19.4)	47 (26.7)	369 (34.1)	0.013	0.238	0.053
Microvascular invasion, present	28 (41.8)	82 (46.6)	614 (56.7)	0.017	0.502	0.012
Adjuvant transarterial chemoembolization, present	22 (32.8)	81 (46.0)	595 (55.0)	<0.001	0.063	0.027

Data are mean ± standard deviation, median (IQR) or N (%).

BCLC, Barcelona Clinic Liver Cancer; CHB, chronic hepatitis B; MAFLD, metabolic dysfunction-associated fatty liver disease.

### Survival Outcomes for Total Population

During a median follow-up of 47 months, 450 of 1325 (34.0%) patients died mainly because of HCC progression and/or hepatic failure. Of the total population (n=1325), 1-year, 3-year, and 5-year overall survival were 87.4%, 68.6%, and 59.8%, respectively (**Supplementary Figure 1A**). One-year, 3-year, and 5-year recurrence-free survival were 57.8%, 39.0%, and 30.7%, respectively (**Supplementary Figure 1B**). Univariable and multivariable analyses for overall and recurrence-free survival are summarized in **Table 2**. MAFLD was independently associated with overall and recurrence-free survival (all *P*<0.05) on multivariable analyses.

**Table 2:** Risk factors of overall and recurrence-free survival.

Variables	Overall survival				Recurrence-free survival			
	Univariable analysis		Multivariable analysis		Univariable analysis		Multivariable analysis	
	Hazard ratio (95% CI)	*P* value	Hazard ratio (95% CI)	*P* value	Hazard ratio (95% CI)	*P* value	Hazard ratio (95% CI)	*P* value
Counterparts								
CHB	1.00		1.00		1.00		1.00	
MAFLD	0.24 (0.11–0.50)	<0.001	0.38 (0.18–0.80)	0.011	0.44 (0.29–0.68)	<0.001	0.54 (0.35–0.83)	0.005
CHB/MAFLD	0.76 (0.57–1.01)	0.061	1.10 (0.79–1.53)	0.555	0.74 (0.58–0.93)	0.010	0.88 (0.68–1.14)	0.328
Gender								
Male	1.00				1.00			
Female	0.76 (0.57–1.02)	0.066			0.78 (0.62–0.98)	0.033		
Age, yr	0.99 (0.98–1.00)	0.126			0.99 (0.98–1.00)	0.002		
BMI, kg/m2								
<23	1.00		1.00		1.00		1.00	
≥23	0.73 (0.61–0.89)	0.002	0.89 (0.72–1.11)	0.294	0.85 (0.73–0.98)	0.029	1.00 (0.85–1.19)	0.968
Smoking								
Absent	1.00		1.00		1.00		1.00	
Present	1.21 (1.00–1.46)	0.047	1.05 (0.86–1.27)	0.643	1.21 (1.04–1.41)	0.014	1.12 (0.96–1.31)	0.148
Hypertension								
Absent	1.00				1.00			
Present	0.86 (0.62–1.20)	0.371			0.67 (0.50–0.89)	0.006		
T2DM								
Absent	1.00				1.00			
Present	0.81 (0.57–1.14)	0.227			0.88 (0.66–1.17)	0.384		
Hepatic steatosis								
Absent	1.00				1.00			
Present	0.68 (0.54–0.85)	0.001			0.71 (0.59–0.85)	<0.001		
Liver cirrhosis								
Absent	1.00				1.00			
Present	0.90 (0.74–1.10)	0.297			0.94 (0.80–1.10)	0.452		
Child-Pugh grade								
A	1.00				1.00			
B	1.38 (0.98–1.94)	0.065			1.18 (0.87–1.60)	0.285		
Triglycerides, mmol/L	0.82 (0.68–0.99)	0.035			0.86 (0.74–0.99)	0.034		
HDL, mmol/L	0.77 (0.58–1.04)	0.087			1.08 (0.86–1.37)	0.498		
Alpha fetoprotein, ng/ml								
<400	1.00		1.00		1.00		1.00	
≥400	1.67 (1.38–2.00)	<0.001	1.17 (0.96–1.41)	0.114	1.73 (1.49–2.00)	<0.001	1.29 (1.11–1.51)	0.001
Platelet count, x10^9^/L								
<100	1.00				1.00			
≥100	0.73 (0.52–1.01)	0.060			0.91 (0.69–1.21)	0.531		
Prothrombin time	1.14 (1.06–1.22)	<0.001	1.00 (0.99–1.00)	<0.001	1.11 (1.05–1.17)	<0.001		
Total bilirubin, μmol/L	1.00 (1.00–1.01)	0.004			1.00 (1.00–1.00)	0.336		
Albumin, g/L	0.96 (0.94–0.98)	<0.001			0.97 (0.95–0.98)	<0.001		
Prealbumin, g/L	0.99 (0.99–1.00)	<0.001			1.00 (0.99–1.00)	<0.001		
ALT, U/L	1.00 (1.00–1.00)	0.781			1.00 (1.00–1.00)	0.050	1.00 (1.00–1.00)	0.038
AST, U/L	1.00 (1.00–1.01)	<0.001			1.01 (1.00–1.01)	<0.001	1.01 (1.00–1.01)	<0.001
Tumor size, cm								
≤5	1.00		1.00		1.00		1.00	
>5	2.61 (2.10–3.24)	<0.001	1.41 (1.11–1.80)	0.005	2.18 (1.85–2.56)	<0.001	1.30 (1.08–1.56)	0.005
Tumor number								
Single	1.00		1.00		1.00		1.00	
Multiple	1.44 (1.17–1.77)	0.001	1.01 (0.77–1.32)	0.949	1.45 (1.22–1.71)	<0.001	1.05 (0.85–1.31)	0.629
Resection								
Minor	1.00		1.00		1.00		1.00	
Major	2.23 (1.85–2.69)	<0.001	1.22 (0.99–1.51)	0.060	1.86 (1.60–2.16)	<0.001	1.02 (0.86–1.21)	0.825
Macrovascular invasion								
Absent	1.00		1.00		1.00		1.00	
Present	3.26 (2.66–3.99)	<0.001	1.57 (1.07–2.28)	0.019	2.66 (2.23–3.17)	<0.001	1.31 (0.96–1.79)	0.083
Microvascular invasion								
Absent	1.00		1.00		1.00		1.00	
Present	2.85 (2.32–3.51)	<0.001	2.14 (1.72–2.66)	<0.001	2.16 (1.84–2.52)	<0.001	1.54 (1.30–1.81)	<0.001
Adjuvant TACE								
No	1.00		1.00		1.00		1.00	
Yes	1.69 (1.40–2.06)	<0.001	1.17 (0.96–1.43)	0.125	1.99 (1.70–2.32)	<0.001	1.43 (1.21–1.69)	<0.001

ALT, Alanine aminotransferase; AST, aspartate aminotransferase; BMI, body mass index; CHB, chronic hepatitis B; HDL, High-density lipoprotein; MAFLD, metabolic dysfunction-associated fatty liver disease; T2DM, type 2 diabetes mellitus; TACE, transarterial chemoembolization.

Postoperative mortality within 30 days after liver resection was observed in two (0.2%) patients in CHB group. However, no postoperative mortality was observed in other two groups. MAFLD group (4.5%, 3/67) had the lowest major morbidity than CHB group (7.9%, 85/1082) and CHB/MAFLD group (10.2%, 18/176) (all *P*>0.05).

**Figure 2:**
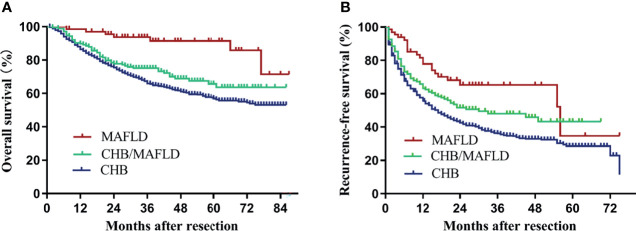
Kaplan–Meier analysis of overall and recurrence-free survival for each counterparts. **(A)** Overall survival (MAFLD *vs* CHB, *P*<0.001; MAFLD *vs* CHB/MAFLD, *P*=0.002; CHB/MAFLD *vs* CHB, *P*=0.059), **(B)** recurrence-free survival (MAFLD *vs* CHB, *P*<0.001; MAFLD *vs* CHB/MAFLD, *P*=0.038; CHB/MAFLD *vs* CHB, *P*=0.010). CHB, chronic hepatitis B; MAFLD, metabolic dysfunction-associated fatty liver disease.

Median follow-up for patients in the MAFLD, CHB/MAFLD, and CHB group were 46 (range, 7-87), 44 (range, 2-86), and 47 (range, 1-86) months, respectively. MAFLD patients had significantly higher overall and recurrence-free survival than CHB/MAFLD patients (*P*=0.002, **Figure 2A**; *P*=0.038, **Figure 2B**) or CHB patients (*P*<0.001, **Figure 2A**; *P*<0.001, **Figure 2B**). In addition, CHB/MAFLD patients had higher overall (*P*=0.059; **Figure 2A**) and recurrence-free survival than CHB patients (*P*=0.010; **Figure 2B**).

### Survival Outcomes Between MAFLD and CHB Patients After Propensity Score Matching

**Figure 3:**
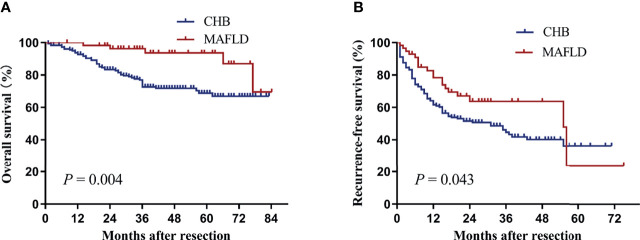
Kaplan–Meier analysis of overall **(A)** and recurrence-free survival **(B)** for the MAFLD and CHB counterparts after propensity score matching. CHB, chronic hepatitis B; MAFLD, metabolic dysfunction-associated fatty liver disease.

Baseline clinical characteristics between MAFLD (n=58) and CHB (n=180) groups were comparable after propensity score matching (**Table 3**). MAFLD patients had significantly higher overall and recurrence-free survival than CHB patients (*P*=0.004, **Figure 3A**; *P*=0.043, **Figure 3B**).

**Table 3:** Baseline demographic and clinical characteristics of patients with MAFLD or CHB.

Variables	Before propensity score			After propensity score		
	MAFLD, n=67 (%)	CHB, n=1082 (%)	*P* value	MAFLD, n=58 (%)	CHB, n=180 (%)	*P* value
Gender, female	10 (14.9)	151 (14.0)	0.824	8 (13,8)	25 (13.9)	1.000
Age, year	60 (52,66)	49 (41,56)	<0.001	57 (50,64)	58 (50,64)	0.527
Smoking, present	20 (29.9)	408 (37.7)	0.197	19 (32.8)	72 (40.0)	0.354
Liver cirrhosis, present	34 (50.7)	771 (71.3)	<0.001	34 (58.6)	106 (58.9)	1.000
Child-Pugh grade B	4 (6.0)	83 (7.7)	0.610	4 (6.9)	16 (8.9)	0.789
Alpha fetoprotein, ≥400 ng/ml	16 (23.9)	457 (42.2)	0.003	15 (25.9)	56 (31.1)	0.511
Platelet count, <100x10^9^/L	1 (1.5)	88 (8.1)	0.048	1 (1.7)	5 (2.8)	1.000
Prothrombin time	12.1 (11.5, 12.8)	12.8 (12.1,13.7)	<0.001	12.1 (11.5,12.7)	12.3 (11.7,13.0)	0.094
Total bilirubin, μmol/L	13.4 (9.9, 18.4)	13.6 (10.1, 18.3)	0.846	13.5 (9.8,19.1)	12.7 (9.6,17.8)	0.465
Albumin, g/L	39.6 (37.0,42.5)	38.8 (35.8,42)	0.104	39.2 (36.5,41.5)	39.3 (35.2,42.8)	0.905
Prealbumin, g/L	222 (183,257)	171 (130,213)	<0.001	213 (175,252)	198 (148,238)	0.051
Alanine aminotransferase, U/L	26.0 (18.5,35.0)	37.0 (26.0,54.0)	<0.001	28.0 (18.8,38.3)	30.5 (23.0,42.0)	0.077
Aspartate aminotransferase, U/L	28.0 (23.0,36.5)	41.0 (31.0, 61.0)	<0.001	26.0 (17.0,38.0)	38.0(24.0,42.0)	0.074
Tumor size, >5 cm	33 (49.3)	667 (61.6)	0.044	28 (48.3)	92 (51.1)	0.764
Tumor number, multiple	7 (10.4)	243 (22.5)	0.021	7 (12.1)	18 (10.0)	0.806
Macrovascular invasion, present	7 (10.4)	197 (18.2)	0.107	7 (12.1)	26 (14.4)	0.674
BCLC stage			0.010			0.792
0/A	52 (77.6)	638 (59.0)		43 (74.1)	125 (69.4)	
B	6 (9.0)	196 (18.1)		6 (10.3)	23 (12.8)	
C	9 (13.4)	248 (22.9)		9 (15.5)	32 (17.8)	
Major hepatectomy	13 (19.4)	369 (34.1)	0.013	11 (19.0)	37 (20.6)	0.853
Microvascular invasion, present	28 (41.8)	614 (56.7)	0.017	25 (43.1)	95 (52.8)	0.228
Adjuvant transarterial chemoembolization, present	22 (32.8)	595 (55.0)	<0.001	22 (37.9)	80 (44.4)	0.446

Data are mean ± standard deviation, median (IQR) or N (%).

BCLC, Barcelona Clinic Liver Cancer; CHB, chronic hepatitis B; MAFLD, metabolic dysfunction-associated fatty liver disease.

### Survival Outcomes Between MAFLD Plus CHB/MAFLD and CHB Patients

**Figure 4:**
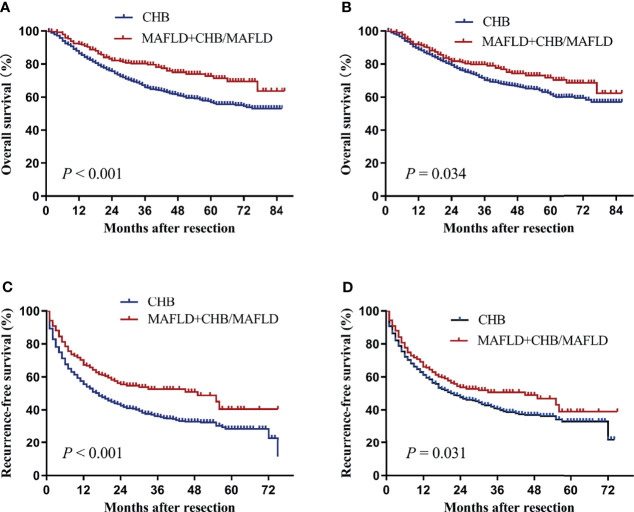
Kaplan–Meier analysis of overall and recurrence-free survival for the MAFLD plus CHB/MAFLD and CHB counterparts. **(A)** Overall survival before propensity matching, **(B)** overall survival after propensity matching, **(C)** recurrence-free survival before propensity matching, **(D)** recurrence-free survival after propensity matching. CHB, chronic hepatitis B; MAFLD, metabolic dysfunction-associated fatty liver disease.

To further explore the difference in prognosis between MAFLD and CHB, patients with MAFLD or CHB/MAFLD were combined in one group. Baseline clinical characteristics of the two groups before and after propensity matching were described in **Table 4**. Patients with MAFLD or CHB/MAFLD had significantly higher overall and recurrence-free survival than CHB patients before or after propensity score matching (all *P*<0.05, **Figure 4**).

**Table 4:** Baseline demographic and clinical characteristics of patients with MAFLD+CHB/MAFLD or CHB.

Variables	Before propensity score			After propensity score		
	MAFLD or CHB/MAFLD, n=243 (%)	CHB, n=1082 (%)	*P* value	MAFLD or CHB/MAFLD, n=232 (%)	CHB, n=698 (%)	*P* value
Gender, female	31 (12.8)	151 (14.0)	0.681	30 (12.9)	100 (14.3)	0.662
Age, year	51 (44, 60)	49 (41, 56)	<0.001	50 (44, 59)	50 (42, 58)	0.207
Smoking, present	76 (31.3)	408 (37.7)	0.065	76 (32.8)	246 (35.2)	0.524
Liver cirrhosis, present	154 (71.3)	771 (71.3)	0.017	152 (65.5)	489 (70.1)	0.219
Child-Pugh grade B	9 (3.7)	83 (7.7)	0.035	9 (3.9)	37 (5.3)	0.485
Alpha fetoprotein, ≥400 ng/ml	67 (27.6)	457 (42.2)	<0.001	67 (28.9)	234 (33.5)	0.196
Platelet count, <100x10^9^/L	9 (3.7)	88 (8.1)	0.020	9 (3.9)	40 (5.7)	0.313
Prothrombin time	12.4 (11.7, 13.3)	12.8 (12.1, 13.7)	<0.001	12.5 (11.7, 13.3)	12.6 (12.0, 13.4)	0.132
Total bilirubin, μmol/L	13.8 (10.3, 17.6)	13.6 (10.1, 18.3)	0.986	13.8 (10.2, 17.8)	13.2(9.9, 17.6)	0.339
Albumin, g/L	39.7 (36.8, 42.4)	38.8 (35.8, 42.0)	0.007	39.6 (36.6, 42.2)	39.2 (36.2, 42.1)	0.223
Prealbumin, g/L	202 (163, 239)	171 (130, 213)	<0.001	197 (162, 236)	190(150, 230)	0.077
Alanine aminotransferase, U/L	35 (25, 52)	37 (26, 54)	0.253	36(25, 53)	34 (25, 51)	0.611
Aspartate aminotransferase, U/L	35 (27, 48)	41 (31, 61)	<0.001	35 (27, 48)	37 (29, 52)	0.067
Tumor size, >5 cm	129 (53.1)	667 (61.6)	0.017	125 (53.9)	407 (58.3)	0.251
Tumor number, multiple	51 (21.0)	243 (22.5)	0.670	50 (21.6)	145 (20.8)	0.852
Macrovascular invasion, present	28 (11.5)	197 (18.2)	0.014	28 (12.1)	102 (14.6)	0.382
BCLC stage			0.008			0.193
0/A	158 (65.0)	638 (59.0)		148 (63.8)	435 (62.3)	
B	51 (21.0)	196 (18.1)		50 (21.6)	127 (18.2)	
C	34 (14.0)	248 (22.9)		34 (14.7)	136 (19.5)	
Major hepatectomy	60 (24.7)	369 (34.1)	0.005	58 (25.0)	207 (29.7)	0.180
Microvascular invasion, present	110 (45.3)	614 (56.7)	0.001	107 (46.1)	351 (50.3)	0.289
Adjuvant transarterial chemoembolization, present	103 (42.4)	595 (55.0)	<0.001	102 (44.0)	339 (48.6)	0.226

Data are mean ± standard deviation, median (IQR) or N (%).

BCLC, Barcelona Clinic Liver Cancer; CHB, chronic hepatitis B; MAFLD, metabolic dysfunction-associated fatty liver disease.

## Discussion

MAFLD increased substantially over the past 20 years (32, 33). Moreover, concurrent diabetes mellitus, overweight/obesity, or hepatic steatosis is associated with increased HCC risk among CHB or HCV patients (34–37). These factors contribute to MAFLD becoming a major cause of HCC in the world. Our understanding of the prognoses of patients with MAFLD-related HCC after liver resection is limited. In agreement with other report (38), MAFLD patients more often had metabolic syndrome, had better synthetic liver function, less often had alpha fetoprotein concentration ≥ 400 ng/mL, tumor size ≥ 5 cm, multinodular, underwent major hepatectomy, and microvascular invasion compared to CHB counterparts at HCC diagnosis. These discrepancies in demographics, comorbidities, and measures of hepatic synthetic function may lead to lower perioperative major morbidity, but higher overall and recurrence-free survival compared to CHB counterparts. MAFLD patients still had a survival advantage after propensity score matching. Moreover, multivariate analyses confirmed the association between MAFLD and overall or recurrence-free survival independent of other clinicopathologic factors.

Among the patients with MAFLD-related HCC, 49.3% were without cirrhosis. This finding reinforces the fact that MAFLD- or NAFLD-related HCC could arise in the absence of cirrhosis in patients with clinically MAFLD or NAFLD (39–42). This phenomenon highlights the importance of HCC screening or surveillance programs for MAFLD or NAFLD patients without cirrhosis. However, the proportion of cirrhosis among the MAFLD patients with HCC was significantly lower than CHB or CHB/MAFLD counterparts, which is consistent with the findings that NAFLD patients with HCC had the lowest proportion of cirrhosis than those with any other etiologies including CHB, HCV and alcoholic liver disease in Eastern or Western centers (3, 38, 39, 41, 43–46). Hence, active HCC surveillance is recommended in MAFLD patients for the early detection of HCC.

Though the low proportion of cirrhosis, major hepatectomy, and better hepatic synthetic function at HCC presentation may translate into lower major morbidity and higher long-term survival after liver resection before propensity matching, MAFLD (or MAFLD plus CHB/MAFLD) patients still had significantly higher overall and recurrence-free survival than CHB patients after propensity matching. Therefore, other essential factor, such as the difference of pathophysiology between MAFLD and CHB (47, 48), may be at the root of the difference in prognoses between the groups. Future studies are expected to compare the difference in prognosis between groups from the perspective of pathophysiology.

MAFLD was originally known as NAFLD. In our cohort, the incidence of MAFLD was 12.9% (243/1887)—similar to that found in other series (41, 45). However, its incidence was higher in series from Korea (52.3%) (46) or USA (33.4%) (44). Though patients with concomitant nonalcoholic steatohepatitis and CHB have more advanced fibrosis, higher HCC risk, and shorter time to development of liver-related outcomes or death compared to patients with CHB alone (35, 37, 49, 50), we did not found CHB/MAFLD patients had more-severe background liver disease or more advanced HCC than CHB patients. On the contrary, CHB/MAFLD counterparts had lower rate of cirrhosis (68.2% *vs* 71.3%), alpha fetoprotein concentration ≥ 400 ng/mL (29% *vs* 42.2%), Barcelona Clinical Liver Cancer stage C disease (14.2% *vs* 22.9%), tumor size ≥ 5 cm (54.5% *vs* 61.6%), microvascular invasion (46.6% *vs* 56.7%), receiving major hepatectomy (26.7% *vs* 34.1%), and better hepatic synthetic function (measured by albumin, prealbumin, and aspartate aminotransferase levels) than CHB counterparts (**Table 1**). A cohort from Korea also found concurrent NAFLD was associated with both better overall and recurrence-free survival in patients with CHB-related HCC than those without NAFLD before adjusting for baseline characteristics (51). However, this survival benefit of the concurrent NAFLD was not significant in multivariable Cox analysis or analysis after propensity score matching (51). In our study, MAFLD counterparts had the highest overall and recurrence-free survival than CHB/MAFLD or CHB counterparts, which was consistent with the findings that NAFLD-related HCC had better long-term survival outcomes compared to non-NAFLD etiologies after liver resection or radiofrequency ablation (36, 38, 41, 44, 45). Our findings was confirmed by propensity score analysis.

The present study has some limitations. First, only patients with HCC underwent potential curative liver resection were included. The clinical impact of MAFLD on the prognoses of HCC patients underwent other therapies, such as transarterial chemoembolization or liver transplantation, could not be evaluated in this study. Second, mild hepatic steatosis may not be detectable by imaging techniques. And fibroscan was not routinely used in the present cohort. Moreover, some cases with hepatic steatosis may not be documented in postoperative histopathological report. As a result, the actual number of MAFLD in the enrolled population may be more than that in the reported population. Third, patients with CHB were included in two groups. Antiviral therapy with nucleos(t)ide analogue would improve overall survival (28). Nevertheless, MAFLD patients still had the best long-term survival compared to CHB or CHB/MAFLD counterparts. Finally, subgroup analysis based on tumor stage or alpha fetoprotein concentration was not performed because of the small sample size of MAFLD group.

## In Conclusion

Patients with HCC in the setting of MAFLD have less-severe background liver disease and better long-term outcomes after potential curative liver resection compared to counterparts with CHB. However, the association between MAFLD and HCC patient prognoses was not fully assessed because the relatively small number of MAFLD patients examined and the potential selection bias. Further well-designed study with a larger sample size is warranted.

## Data Availability Statement

The datasets presented in this article are not readily available because all data were in the article. **Supplementary Material** for this article is available by request to J-HZ. Requests to access the datasets should be directed to J-HZ, zhongjianhong@gxmu.edu.cn.

## Ethics Statement

This study was approved by the Ethics Committees of our hospitals (LW2021042; LW2021005). Written informed consent for participation was not required for this study in accordance with the national legislation and the institutional requirements.

## Author Contributions

J-HZ conceived the study. All authors participated in the acquisition of the data. R-RH and J-HZ analyzed data. LL and J-HZ drafted and revised the manuscript. All authors contributed to the article and approved the submitted version.

## Funding

J-HZ is in part supported by the National Natural Science Foundation of China (82060510), ‘Guangxi BaGui Scholars’ Special Fund (2019AQ20), the Natural Science Foundation of Guangxi Province (2020GXNSFAA159022), and the Guangxi Undergraduate Training Program for Innovation and Entrepreneurship (202110598178, 202110598073). B-DX is in part supported by the “High-level innovation team and outstanding scholar program in Guangxi Colleges and Universities”. X-ML is in part supported by the Self-raised Scientific Research Fund of the Ministry of Health of Guangxi Province (Z20200923).

## Conflict of Interest

The authors declare that the research was conducted in the absence of any commercial or financial relationships that could be construed as a potential conflict of interest.

## Publisher’s Note

All claims expressed in this article are solely those of the authors and do not necessarily represent those of their affiliated organizations, or those of the publisher, the editors and the reviewers. Any product that may be evaluated in this article, or claim that may be made by its manufacturer, is not guaranteed or endorsed by the publisher.

## Supplementary Material

Supplementary Figure 1 Kaplan–Meier analysis of overall **(A)** and recurrence-free survival **(B)** for the total population.
