# Supplementary figures and images for: Outcomes of Liver Resection for Metabolic Dysfunction-Associated Fatty Liver Disease or Chronic Hepatitis B-Related HCC
Source: Front Oncol. 2022 Jan 20;11:783339. doi: 10.3389/fonc.2021.783339 (PMC8810487; doi:10.3389/fonc.2021.783339)

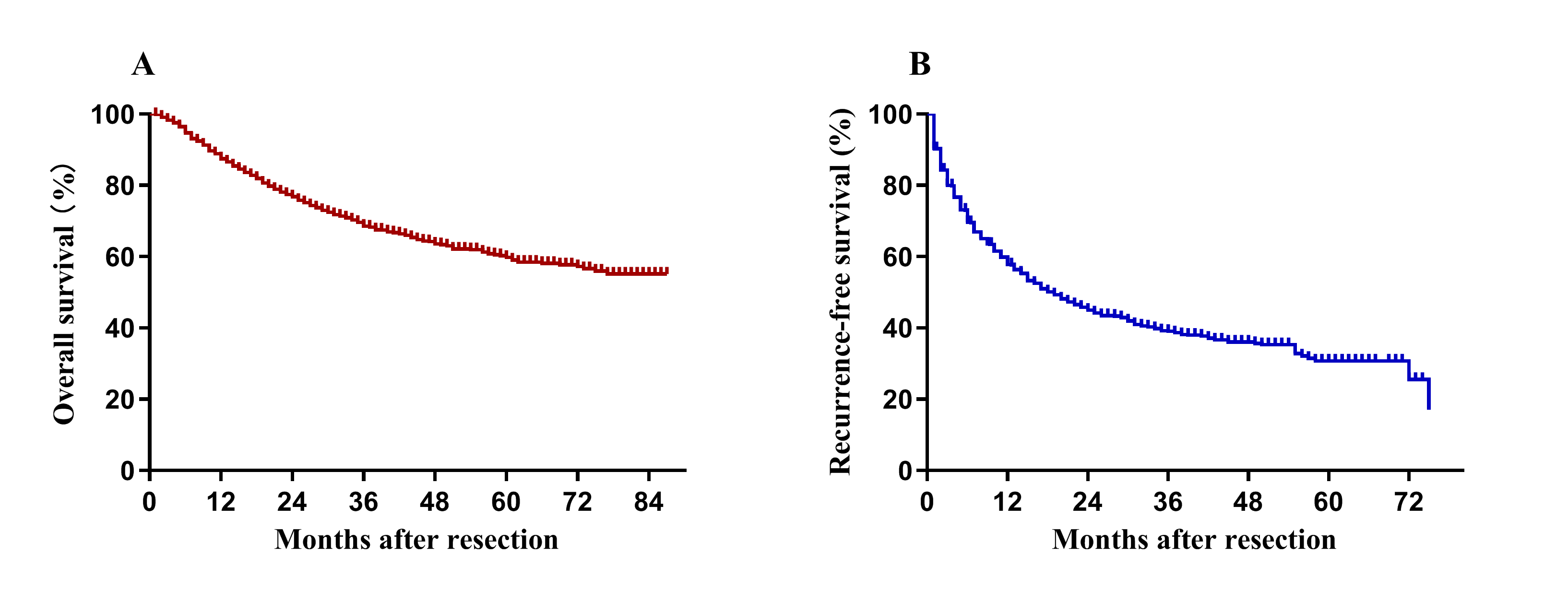

Supplement: Supplementary Figure 1 — Kaplan–Meier analysis of overall (A) and recurrence-free survival (B) for the total population. [file Image_1.tif]
